# Twenty-one days of low-intensity eccentric training improve morphological characteristics and function of soleus muscles of *mdx* mice

**DOI:** 10.1038/s41598-020-79168-3

**Published:** 2021-02-11

**Authors:** Paulo S. Pedrazzani, Tatiana O. P. Araújo, Emilly Sigoli, Isabella R. da Silva, Daiane Leite da Roza, Deise Lucia Chesca, Dilson E. Rassier, Anabelle S. Cornachione

**Affiliations:** 1 Department of Physiological Science, Federal University of São Carlos (UFSCar), São Carlos, Brazil; 2 Department of Kinesiology and Physical Education, McGill University, Montreal, Canada; 3 Department of Neurosciences and Behaviour, Ribeirão Preto Medical School, University of São Paulo, Ribeirão Preto, Brazil; 4 Department of Pathology and Legal Medicine, Ribeirão Preto Medical School, University of São Paulo, Ribeirão Preto, Brazil

**Keywords:** Musculoskeletal system, Biophysics, Physiology

## Abstract

Duchene muscular dystrophy (DMD) is caused by the absence of the protein dystrophin, which leads to muscle weakness, progressive degeneration, and eventually death due to respiratory failure. Low-intensity eccentric training (LIET) has been used as a rehabilitation method in skeletal muscles after disuse. Recently, LIET has also been used for rehabilitating dystrophic muscles, but its effects are still unclear. The purpose of this study was to investigate the effects of 21 days of LIET in dystrophic soleus muscle. Thirty-six male *mdx* mice were randomized into six groups (n = 6/each): *mdx* sedentary group; *mdx* training group-3 days; *mdx* training group-21 days; wild-type sedentary group; wild-type training group-3 days and wild-type training group-21 days. After the training sessions, animals were euthanized, and fragments of soleus muscles were removed for immunofluorescence and histological analyses, and measurements of active force and Ca^2+^ sensitivity of the contractile apparatus. Muscles of the *mdx* training group-21 days showed an improvement in morphological characteristics and an increase of active force when compared to the sedentary *mdx* group. The results show that LIET can improve the functionality of dystrophic soleus muscle in mice.

## Introduction

Duchenne muscular dystrophy (DMD) is a lethal pediatric muscle disorder caused by a mutation in the region Xp 21.2 *(dystrophin gene)* that leads to an absence of dystrophin protein. DMD affects one in every 5000 boys^1^ and causes many deficiencies including an impaired mobility, wheel chair confinement, respiratory and cardiac failure. Dystrophin and the dystrophin-glycoprotein complex have an important role in the sarcolemma membrane integrity during muscle contraction. Lack of dystrophin in muscle fibers leads to mechanical damage, impaired Ca^2+^ homeostasis, increased proteolysis and widespread cellular dysfunction^2–4^, resulting in weakness and loss of muscle function.

There is no cure for DMD, but there are rehabilitation exercise programs aimed to slow the progression of the disease^5^. Although the effects of exercise for DMD are still controversial^6,7^, there is an increasing interest in the potential benefits of physical training, since it is cost-effective and non-invasive. Currently, there is no consensus on the best type, frequency and intensity of exercise to be used with DMD^7–9^. Studies conducted with *mdx* mice, an animal model for DMD, have shown that physical training can improve muscle function without aggravating the disease. Low-intensity training (LIT) in treadmill running improves the overall function of *mdx* muscles, reduces oxidative stress and inflammation, while increasing their mitochondrial capacity^10–12^ and oxidative capacity^13,14^. There are also significant improvements in strength, resistance to fatigue and muscle morphology after 10–16 weeks of treadmill training^15–17^. The most common types of training used in studies with dystrophic mice include running on a flat treadmill^18^, running on wheels, or swimming for long periods of time (10–16 weeks)^15–17,19^.

Eccentric exercise, which can lead to muscle injury^19–22^, has also been shown to partially re-establish some of the cytoarchitectural features in disused muscles, especially when incorporated in a training protocol conducted for long periods of time^23–26^. These studies include muscles that were immobilized and used low-intensity exercise, because it is known that a high-intensity eccentric training can lead to irreversible muscle damage. There are no studies in the literature that address low-intensity eccentric exercise in *dystrophic* muscles for a long period of time. Although dystrophic muscles are more vulnerable to injuries caused by eccentric contractions than healthy muscles^27,28^, many physiological tasks such as walking or sitting involve eccentric contractions.

The purpose of this study was to investigate if low-intensity eccentric training (LIET) applied during a prolonged period of time could improve the contractile function and morphology of the soleus muscle of *mdx* mice. We observed that 21 days of a LIET in treadmill induced positive adaptations in dystrophic muscles, suggesting that it may be a positive strategy to mitigate the effects of DMD in skeletal muscles.

## Results

### Morphological alteration

**Figure 1:**
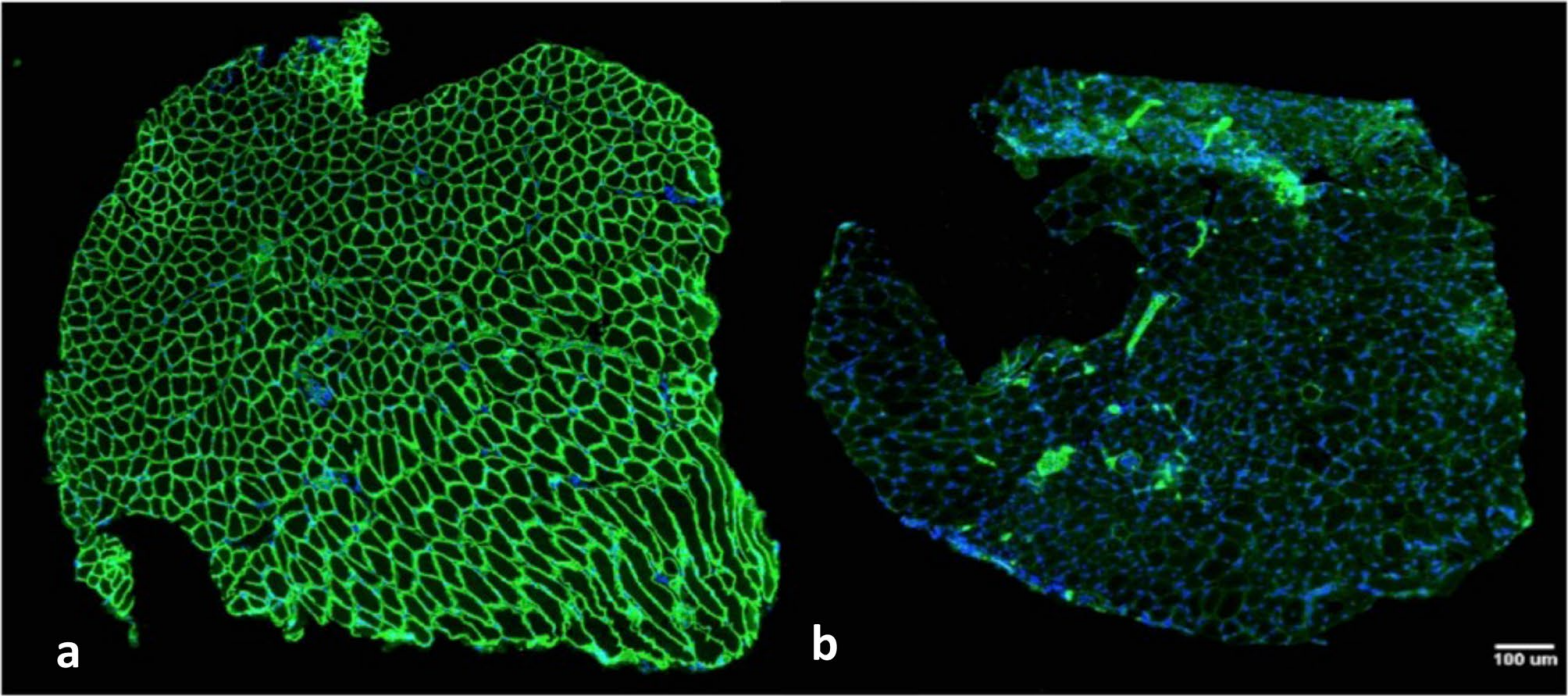
Photomicrographs of soleus muscle immunolabelled by dystrophin antibody. **A** *wt*SED sample, with dystrophin protein shown in green and nucleus is shown in blue (DAPI). **B** *mdx*SED sample, with nucleus shown in blue, without the presence of dystrophin protein. Bars: 100 μm. Abbreviations: wild-type Sedentary group (*wt*SED); *mdx* Sedentary group (*mdx*SED).

Immunofluorescence analysis confirmed the absence of dystrophin in *mdx* mice muscle. Figure 1 shows that dystrophin is present in the sarcolemma of soleus fibers in wild-type mice (green color—Fig. 1A), but is absent in *mdx* mice (Fig. 1B).

**Figure 2:**
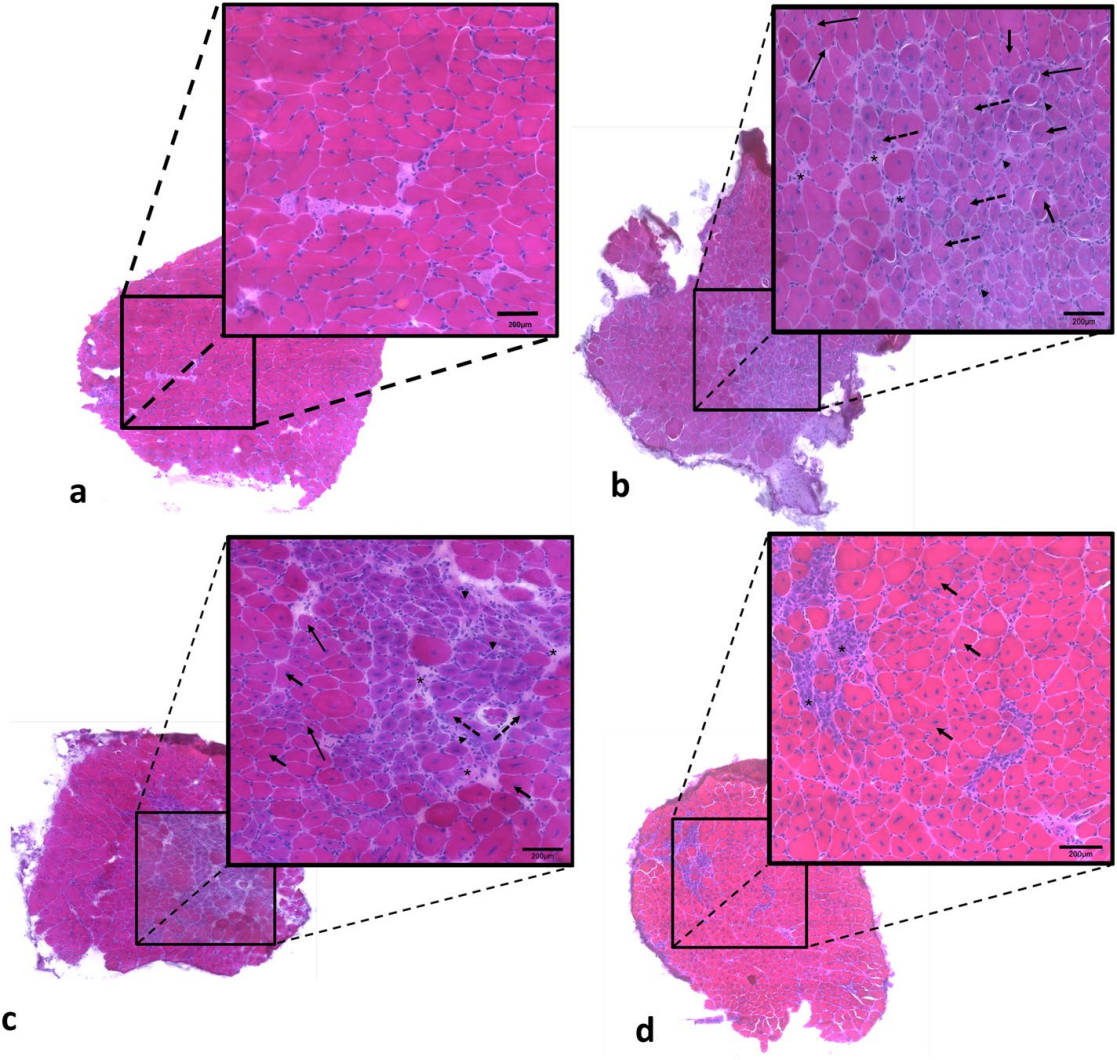
Photomicrographs of soleus muscle stained with hematoxylin and eosin (HE). **A** *wt*SED sample, with polyhedral fibers with peripheral nuclei; **B** *mdx*SED sample, showing variations in fiber size, increased of connective tissue (*), nuclear centralization (thick arrow), splitting (thin arrow), necrosis (arrowhead), cytoplasmic rarefaction (dotted arrow), and basophilic cell (purples cell); **C** *mdx*TR_3_ sample, showing variation in fiber size, increased of connective tissue (*), nuclear centralization (thick arrow), cytoplasmic rarefaction (dotted arrow), and splitting (thin arrow); **D** *mdx*TR_21_ sample, showing less variation in fiber size, in connective tissue (*), and nuclear centralization (thick arrow). Bars: 200 μm. Abbreviations: wild-type Sedentary group (*wt*SED); *mdx* Sedentary group (*mdx*SED); wild-type Training group for 3 days (*wt*TR_3_); *mdx* Training group for 3 days (*mdx*TR_3_); wild-type Training group for 21 days (*wt*TR_21_); and *mdx* Training group for 21 days (*mdx*TR_21_).

The morphological examination of sections from muscles stained by Hematoxylin and Eosin (HE) revealed a homogeneous pattern of muscle fibers in wild-type mice. Photomicrography shows polyhedral fiber and nuclei in the periphery of the fiber (Fig. 2A). The dystrophic muscles from the *mdx*SED group (Fig. 2B) showed a large variation in fiber size, nuclear centralization, basophilic fibers and necrosis. Although we have not quantified the amount of connective tissue in these muscles, which may limit a definitive conclusion, qualitative analysis showed an increase in connective tissue in dystrophic muscles, a result on line with previous studies^29^.

After eccentric low-intensity training during 3-days (*mdx*Tr_3_) the dystrophic muscles showed a dramatic increase in the degree of morphological abnormalities (Fig. 2C). These alterations include an increase in necrosis and basophilic fibers, variation in fiber size and an increase of connective tissue when compared to animals of *mdx*SED group. Notably, the muscles from dystrophic animals trained during 21 days (*mdx*Tr_21_) showed an improvement in their morphological characteristics. This group presented moderate abnormality when compared with *mdx*Tr_3_. There is a significant decrease of basophilic fibers, degree of necrosis and connective tissue (Fig. 2D). These muscles of *mdx*Tr_21_ group still showed a high-centralized nucleus and with a more homogenous trophism of the fibers, leading to a small variation in fiber sizes (Fig. 2D).

### Minimal Feret’s diameter of the different fiber types

The absence of dystrophin protein caused a significant alteration in the trophism in muscle fibers of *mdx* mice. The muscle of *mdx*SED group showed a significant increase in the diameter of unmixed (FTI, FTIIA and FTIID) and hybrids fibers (FTIC and FTIIAD) when compared to animals of the wild-type group (*mdx*SED vs.* wt*SED; *p* < 0.05). The results of the minimal Feret’s diameter of the different fiber types are shown in Table 1.

**Table 1:** Mean of minimal Feret’s diameter (μm) and the respective 95% confidence intervals in soleus fibers type I, type IIC, type IIA, type IIAD and type D fibers in the different groups studied.

	*wt*SED	*mdx*SED	*wt*TR_*3*_	*mdx*TR_*3*_	*wt*TR_*21*_	*mdx*TR_*21*_
FTI	32.10*	39.92	30.72^†^	27.67*	32.6ˆ	31.68*^¤^
	31.7–32.4	39.01–40.83	30.34–31.1	27.16–28.18	32.13–33.10	31.17–32.20
FTIIC	23.65*	33.36	23.09	24.08*	25.28	27.35*^¤^
	23.1–24.20	32.19–34.54	22.39–23.80	23.61–24.54	24.14–26.42	26.67–28.03
FTIIA	25.87*	36.86	25.02	27.81*	29.76ˆ^†^	34.16*^¤^
	25.65–26.1	36.03–37.63	24.78–25.26	27.48–28.14	29.39–30.13	33.64–34.68
FTIIAD	27.52*	39.99	27.25	27.94*	31.75ˆ^†^	33.37*^¤^
	26.87–28.16	37.83–42.16	26.57–27.94	26.92–28.96	30.73- 32.76	31.73–35.01
FTIID	30.65*	40.76	29.46	38.29*	33.88ˆ^†^	39.72
	29.89–31.41	38.74–42.77	28.65–30.28	36.62–39.97	32.94–34.82	37.62–41.83

*p < 0.05 compared to *mdx*SED; ^†^*p* < 0.05 compared *to wt*SED; ¤*p* < 0.05 compared to *mdx*TR_*3*_; ˆcompared to *wt*TR_3_*.*

The effects of LIET on the trophism of all conditions investigated in this study are summarized in Table 1. LIET was effective to improve the trophism of muscle fibers in the *mdx* mice. The 3-days of training already caused a reduction in the diameter of the dystrophic muscles, while causing only a reduction in the FTI fibers the wild-type trained. The maintenance of the training for 21 days caused an increase in the diameter of all fiber types of the muscles from dystrophic animals (*mdx*TR_21_) compared to animals trained for 3 days (*mdx*TR_3_). Nevertheless, the values were still lower when compared to sedentary dystrophic animals. Similar results were observed in the wild-type group that trained 21 days when compared to the wild-type group that trained for 3 days.

### Proportion of the different fiber types

**Figure 3:**
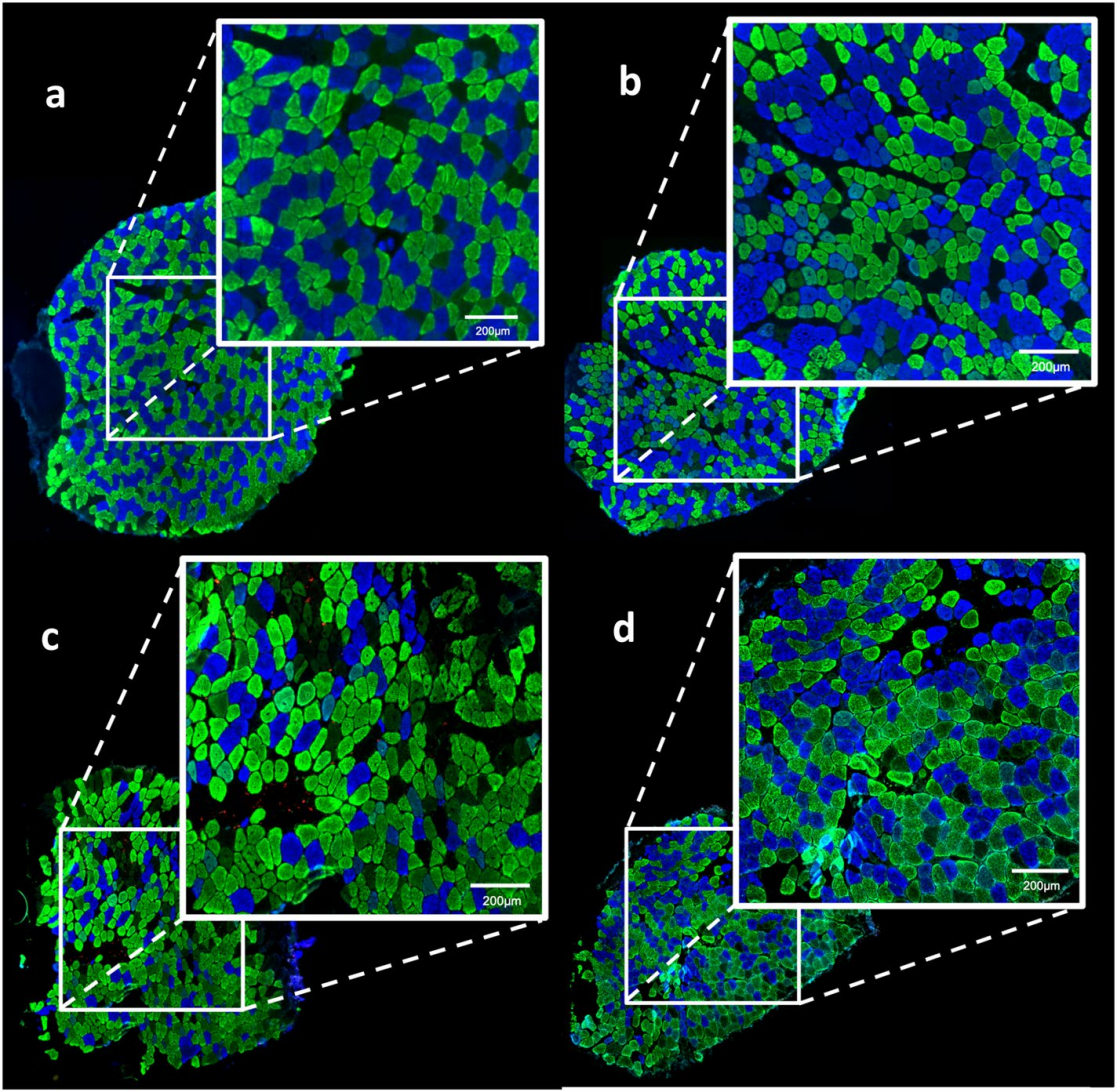
Photomicrographs of soleus muscle immunolabelled by different myosin heavy chain (MHC) antibodies. MHCI (FTI) stained in blue; MHCIIA (FTIIA) stained in green; MHCIID (FTIID) did not stain (black color) and MHCIIB (FTIIB) stained in red. There are not FTIIB in soleus muscle. **A** *wt*SED sample, showing a homogeneous distribution between FTI (blue color) and FTIIA (green color); **B** *mdx*SED sample, showing a grouping of fibers; **C** *mdx*TR_3_ sample, showing heterogeneity in fiber distribution with an increase of FTIIA (green fibers) and FTIID (black fibers) and a decrease of FTI (blue fibers). **D** *mdx*TR_21_ sample, showing a larger degree of homogeneity between FTI (blue fibers) and FTIIA (green fibers). Bars: 200 μm. Abbreviations: wild-type Sedentary group (*wt*SED); *mdx* Sedentary group (*mdx*SED); wild-type Training group for 3 days (*wt*TR_3_); *mdx* Training group for 3 days (*mdx*TR_3_); wild-type Training group for 21 days (*wt*TR_21_); and *mdx* Training group for 21 days (*mdx*TR_21_).

**Figure 4:**
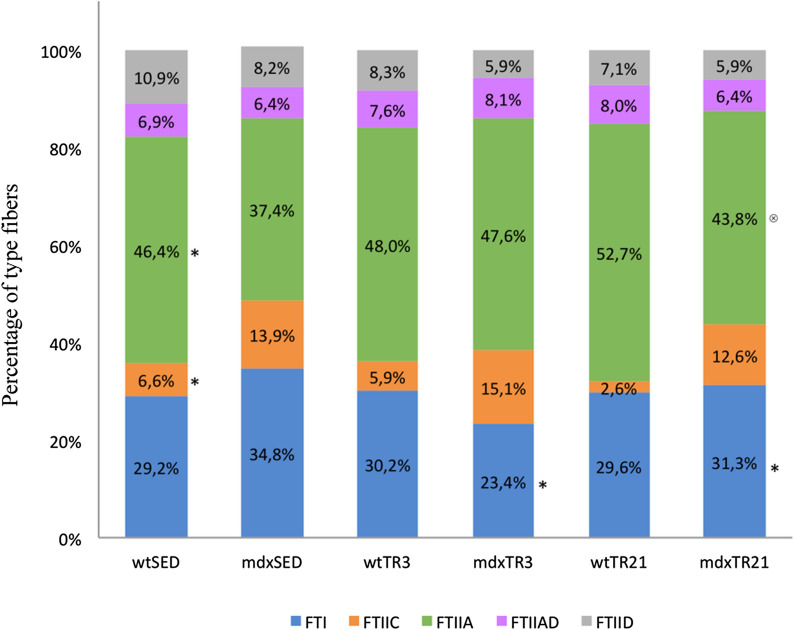
Percentage measurement of type I, IIC, IIA, IIAD and IID of muscle fibers in all groups studied. ^*^p < 0,05 compared to *mdx*SED, ^⊗^*p* < 0.05 compared to *mdx*TR_3._ Abbreviations: wild-type Sedentary group (*wt*SED); *mdx* Sedentary group (*mdx*SED); wild-type Training group for 3 days (*wt*TR_3_); *mdx* Training group for 3 days (*mdx*TR_3_); wild-type Training group for 21 days (*wt*TR_21_); and *mdx* Training group for 21 days (*mdx*TR_21_).

Morphometrics alterations were observed through analysis of the slides processed by immunofluorescence for different MHC isoforms. Figure 3A,B show slides from *wt*SED and *mdx*SED, respectively. Figure 3A shows a homogeneous distribution between FTI (blue color) and FTIIA (green color) fibers while Fig. 3B shows grouping of fiber in the fascicles, i.e., typing group. Figure 4 illustrates the distribution of fiber types in all groups tested in this study. As expected, the absence of dystrophin protein implied a significant reduction in the number of FTIIA fibers with a concomitant increased of FTIIC fibers in sedentary animals.

The short-term training applied to *mdx* mice (*mdx*TR_3_) did not cause a significant alteration in the proportion of different type fibers. Although it the *mdx*TR_3_ showed a small increase of the number in FTIIA fibers and a reduction in FTI fibers (Fig. 4), the difference was not statistically significant. Figure 3C illustrates a predominance of glycolytic FTIIA (green color) in the *mdx*TR_3_ group.

Training of *mdx* mice for 21 days caused a reduction of oxidative fibers (FTI) compared to the *mdx* sedentary group (*mdx*SED vs.* mdx*TR_21_; *p* < 0.05). There was not a difference between the proportions of FTI in both trained groups (*mdx*TR_3_ vs.* mdx*TR_21_; *p* > 0.05), but there was a slight increase in these fibers (*mdx*TR_3_ *vs mdx*TR_21_; p < 0.05). Figure 3D shows a large homogeneity between FTI and FTIIA fibers after a long period of training. The use of low-intensity eccentric exercise did not affect the distribution of different fiber types in healthy animal muscles (Fig. 4).

### Total force of the single cell

**Figure 5:**
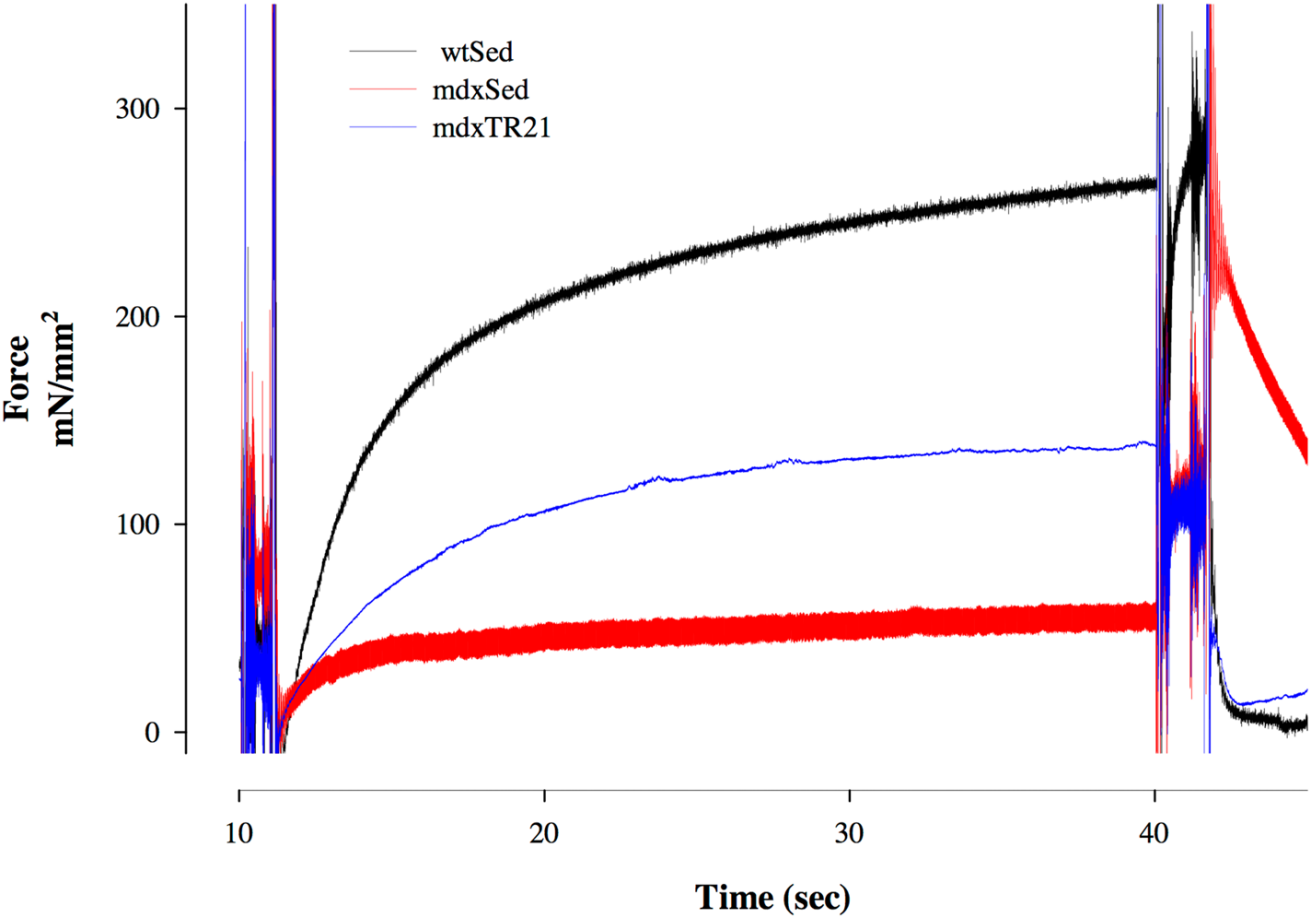
Contractions produced by a single fiber dissected from the soleus muscle from a mouse from *wt*SED (black line), *mdx*SED (red line) or *mdx*TR_21_ (blue line) groups. Abbreviations: wild-type Sedentary group (*wt*SED); *mdx* Sedentary group (*mdx*SED); and *mdx* Training group for 21 days (*mdx*TR_21_).

Figure 5 shows the force traces collected during contractions developed by singles fibers dissected from mice from *wt*SED, *mdx*SED and *mdx*TR_21_. The isometric contractions were recorded during experiments in which fibers were maximally activated (pCa^2+^ 4.5) at an initial sarcomere length of 2.5 μm. The forces produced during maximal activation were lower in *mdx* fibers than wild-type fiber (black trace). After 21 days of low-intensity eccentric training the total force increased significantly in the *mdx* fibers, but was still lower than the force produced by wild-type fibers (Fig. 5). The force values were significantly different among the *wt*SED (241.5 ± 33.8), *mdx*SED (mean: 67.20 ± 17.24) and *mdx*TR_21_ (106.5 ± 8.82) groups.

**Figure 6:**
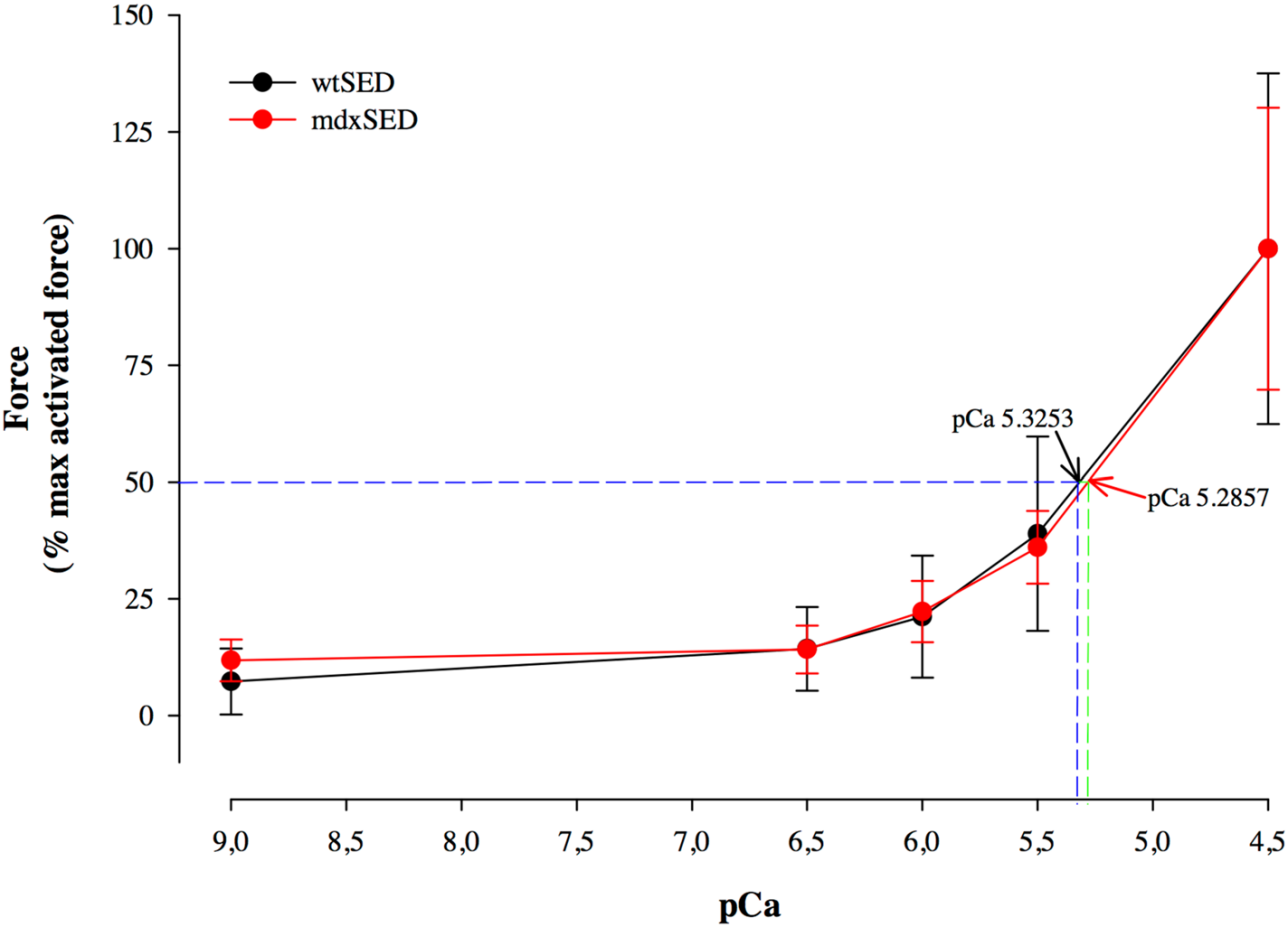
Force-pCa^2+^ relation for the *wt*SED and the *mdx*SED groups. There was no difference in Ca^2+^ sensitivity, as the value for pCa^50^ was not different between the control (mean 5.35 ± 0.08) and *mdx* (mean 5.41; ± 0.13) groups. Abbreviations: wild-type Sedentary group (*wt*SED); *mdx* Sedentary group (*mdx*SED).

We also tested the force responses to different levels of Ca^2+^ activation, to construct a force-pCa curve and evaluate the Ca^2+^ sensitivity of the contractile apparatus (Fig. 6). A difference in Ca^2+^ sensitivity (measured by the pCa^50^) was not observed among groups, i.e., Ca^2+^ sensitivity was not impaired in the *mdx* fibers investigated in this study (Fig. 6).

## Discussion

The results of this study demonstrate that LIET improved the general histology, trophism, the overall distribution of different fiber types and the contractile force of *mdx* mice. These findings suggest that a well-controlled LIET is a viable therapeutic model for improving muscle function in DMD.

Physical exercise has been studied as a therapeutic strategy to delay the progression of DMD. However, physical exercise must be carefully planned because it can also accelerate a degenerative process in DMD muscles. High-intensity exercise, for example, can increase muscle damage and degeneration^18,30^. Although our findings showed a worsening of the phenotype of *mdx* animals trained for 3 days with LIET, this result was reverted with 21 days of training. It has been shown that eccentric exercise when applied for a prolonged period may improve the muscle morphological characteristics^14,31^.

This study confirmed some of the main alterations induced by the absence of dystrophin in soleus muscle: nuclear centralization, splitting, necrosis, increased connective tissue and basophilic cells. The centralization observed in the dystrophic muscle of *mdx* mice is a result of muscle degeneration and regeneration^32^. During repair of skeletal muscle tissues, satellite cells, which are precursors of myogenesis, are activated and proliferate to the site of the lesion where they will merge into the focus of the lesion and subsequently differentiate into myoblasts. These newly repaired cells present the centralized nucleus until the maturation occurs, with subsequent migration of the nucleus to the periphery^33^. Other cytoarchitectural changes such as basophilia and splitting are characteristic of cell membrane damage and Ca^2+^ influx into the cytoplasm of the dystrophic cell^34^, which triggers degenerative reactions that increase the inflammation, contributing to chronic damage and degeneration of dystrophic cells^35^. The degenerative process culminates with increased connective tissue that can lead to fibrosis and impairment of muscle function^32,36,37^.

There was an increase in minimal Feret’s diameter of fibers of the *mdxSED* animals when compared to *wtSED* animals, as previously observed^14,38–40^. The successive degeneration/regeneration processes that occur in the dystrophic fiber exacerbate the inflammatory process in the cytosol, increasing the cell volume^41–43^. After 21 days of training, the soleus muscle cells of the *mdx* animals reduced their volume compared to the *mdx* sedentary; in it has been shown that low-intensity eccentric training applied during long period reduces inflammatory processes^44^ and reduce the cross-section area (CSA) of *mdx* muscle fibers^15^.

The *wt*SED animals presented a homogeneous distribution between type I and type IIA fibers, as previously shown^15,27^. The absence of dystrophin promoted an imbalance in this distribution, as the proportion of FTIIA fibers was reduced while the proportion of FTI fibers was increased in *mdx*SED animals^27,45,46^. We observed that the LIET applied during a short period of training (3-days) reduced the number of FTI fibers. However, with the maintenance of the training for 21 days, the proportion of these oxidative fibers was increased, concomitant with a reduction in FTIIA fibers, in agreement with previous studies^6,47–49^. It is likely that low-intensity training promotes adaptations in the mitochondria thus increasing the oxidative capacity of the fibers and the resistance to fatigue.

The most striking symptom of DMD is the loss of muscle strength due to progressive degeneration of muscle fibers. In this condition, non-contractile tissues, such as adipose and connective tissue, replace the contractile tissue. Our results from experiments conducted with single fibers showed that DMD reduced the specific force of *mdx* muscle, confirming previous results^27,50–54^. The reduction in force in the *mdx* mice could be explained by a reduction of the sensitivity of the contractile system to Ca^2+^, which would lead to a decrease in force produced at a given level of activation^55,56^. However, our results do not show an altered difference in Ca^2+^ sensitivity in fibers dissected from *mdx* mice, as previously observed^57–59^. Therefore, the decrease in force in mdx fibers may be a result of dysfunction in contractile proteins. In this regard, there are conflicting results in the literature. Canepari, et al.^52^ observed an impairment of the myosin function in dystrophic muscles that was attributed to post-translational modifications, which could determine a change in its enzymatic or mechanical properties of myosin. On the other hand, Bates, et al.^60^ did not observe a significant reduction in myosin activity or cross-bridge kinetics in mdx mice. The difference in results is difficult to explain, but it may be associated with differences in experimental protocols, and with the age of the *mdx* mice. In this study, we used 7-week-old mice, an age in the peak phase of the lesion, presenting many cells in degeneration/regeneration, as discussed earlier.

We are not aware of other studies investigating the effects of LIET on contractile properties of single fibers from dystrophic muscles. However our results are on line with studies showing an improvement in the forelimb and hindlimb strength of mdx mice after long periods of low intensity training on a flat treadmill^11,44,61^. There are studies showing an improvement in fatigue resistance and oxidative capacity after 12 weeks of low-intensity training^47^.

## Conclusion

LIET improved the morphological and functional characteristics of the dystrophic soleus muscle of mice by reducing cell degeneration, improving trophism and distribution of different types of fibers and increasing the contractile force of muscle fibers. LIET may be an important method to be used for rehabilitation of dystrophic muscles, with potentially far-reaching implications for the treatment of the disease.

## Methods

### Ethics statement

This study was conducted in accordance with the Ethical Principles in Animal Research, adopted by the National Council for the Control of Animal Experimentation (CONCEA, Brazil) and was approved by the Ethics Committee on Animal Use in Ribeirão Preto Medical School (Brazil) (protocol number 173/2013).

### Animals

Thirty-six male mice were used in this study. Eighteen *mdx* mice (C57BL/10-Dmd^mdx^/PASUnib; body weight 17.37 g; ± 0.48) and eighteen wild-type mice (background: C57BL/10; body weight 19.16 g; ± 0.39) were acquired from CEMIB (Multidisciplinary Center for Biological Investigation on Laboratory Animal Science, UNICAMP, Campinas, Brazil). The animals were bred in the Pasteur Institute (Paris, France).

The mice were maintained in cages on a light–dark cycle (12 h/12 h) at 22ºC and supplied with water and food ad libitum. The *mdx* mice were randomized into three groups (n = 6 for each group): Sedentary group (*mdx*SED), Training group—3 days (*mdx*TR_3_) and Training group—21 days (*mdx*TR_21_). Wild-type mice were similarly randomized into three groups (n = 6 for each group): *wt*SED; *wt*TR_3_ and *wt*TR_21_.

Training started when the animals were 6-weeks old, because at this age severe morphological alterations and signals of degeneration and/or regeneration are detected in dystrophic muscles^62^. The exercise protocol was performed during three days per week (Monday, Wednesday and Friday) for 3 days (1 week) or 21 days (7 weeks) of training. Therefore, at the end of training the animals of *mdx*TR_3_ and *wt*TR_3_ groups were 7-week-old and the animals of *mdx*TR_21_ and *wt*TR_21_ were 13-week-old. The sedentary animals (*mdx*SED and *wt*SED) were 7-week-old.

### Low-intensity training

#### Eccentric protocols

The training protocols were performed on a declined (-16º) treadmill (EP 132C; Insight, Ribeirão Preto, Brazil). Before the training period, all animals underwent a period of adaptation 3 times/week (Monday, Wednesday, Friday), in which they run for 2 min, at a speed of 7 m/min. During the training period, the animals were also exercised 3 times/week (Monday, Wednesday, Friday), but for 10 min/day at a speed of 10 m/min.

### Histology and Immunofluorescence

The soleus muscle of all animals was excised and frozen in liquid nitrogen for histological and immunofluorescence examination. Slides were prepared by sectioning the muscles (6 μm in thickness) using a cryostat (Leica CM 1860, Leica Mikrosysteme Vertrieb GmbH, Wetzlar, Germany) at – 25 °C. Hematoxylin and eosin (H&E, MERCK, Darmstadt, Germany) stains were used to analyze morphological features through light microscopy: nucleus centralized, basophilic cells, and necrosis^23,63^.

Immunofluorescence was applied to quantify different isoforms of myosin heavy chain (MHC) and dystrophin proteins. The slides with frozen sections were blocked with M.O.M. (mouse on mouse—Vector Laboratories, Burlingame, USA). Section were incubated in primary antibodies for dystrophin (MANDYS1, 1:5; DSHB—Developmental Studies Hybridoma Bank, Iowa, USA), MHC type 1 (BA-D5, 1:50; DSHB, Iowa, USA), MHC type 2A (SC-71; 1:100; DSHB, Iowa, USA), MHC type 2D (6H1, 1:100; DSHB, Iowa, USA) and MHC type 2B (BF-F3, 1:50; DSHB, Iowa, USA) in 1% of BSA (Bovine Serum Albumin—Sigma Aldrich, San Luis, Missouri, USA) for 30 min at 37 ºC. The slides were washed with PBS and incubated in secondary antibodies (Alexa Fluor 488, 1:200; Alexa Fluor 594, 1:200; DyLight 405, 1:200; Jackson Immuno Research, Pennsylvania, USA). Sections were mounted in polyvinyl alcohol solution or in prolong gold with DAPI. The slides were analyzed with Image X-Press (Molecular Devices, San Jose, CA, USA) and fluorescence illumination. The minimal Feret’s diameters—the minimum distance of the parallel tangents in opposing borders of the muscle fiber^40^—were measured using Image J software (version 1.50e, NIH, USA). The proportion of fiber types was quantified using whole muscle sections.

### Contractile measurements of single fiber

The muscles were dissected, tie to wood sticks, and chemically permeabilized following standard procedures^64–66^. Briefly, the muscle samples were incubated in rigor solution (50 mM Tris, 100 mM NaCl, 2 mM KCl, 2 mM MgCl_2_, and 10 mM EGTA pH7.0, Sigma Aldrich, San Luis, Missouri, USA) for 4 h, after which they were transferred to a rigor-glycerol (50:50) solution for 15 h. Then, the samples were placed in a fresh rigor-glycerol (50:50) solution with a cocktail of protease inhibitors (Roche Diagnostics, Basel, Switzerland) and stored in a freezer −20 ºC for at least 7 days.

#### Single cell force measurements

On the day of experiment, single fibers were carefully dissected in relaxing solution, and gripped at their ends with T-shaped clips made of aluminum foil. The fiber was transferred to a temperature-controlled chamber (802 D, Aurora Scientific, Aurora, Ontario, Canada) where it was attached between a force transducer (403A, Aurora Scientific, Aurora, Ontario, Canada) and a length controller (322C, Aurora Scientific, Aurora, Ontario, Canada).

Before the start of each experiment, the average sarcomere length (SL) was measured in relaxing solution (100 mM KCl, 2 mM EGTA, 20 mM imidazole, 4 mM ATP, and 7 mM MgCl2, Sigma-Aldrich, San Luis, Missouri, USA) using a high-speed video system (HVSL, Aurora Scientific 901B, Aurora, Ontario, Canada). Images from a selected region of the fibers were used to calculate the SL by fast Fourier transform (FFT) analysis based on the striation spacing produced by dark and light bands of myosin and actin filaments. The fiber diameter and length were measured using a CCD camera (Go-3, QImaging; pixel size: 3.2 μm × 3.2 μm, Aurora Scientific, Aurora, Ontario, Canada), and the cross- sectional area was estimated assuming a circular geometry. Fibers were activated with different Ca^2+^ concentration (pCa 4.5; 5.5; 6.0; 6.5; 9.0) (20 mM imidazole, 14.5 mM creatine phosphate, 7 mM EGTA, 4 mM MgATP, 1 mM free Mg^2+^, and free Ca^2+^ ranging from 1 nM (pCa^2+^ 9.0) to 32 M (pCa^2+^ 4.5) (Sigma-Aldrich, San Luis, Missouri, USA) at an intitial SL of 2.5 μm. The active force produced during the experiments was measured after force stabilized during the contractions.

### Statistical analysis

All contractile data were compared among groups using a two-way analysis of variance (ANOVA) for repeated factors. When significant changes were observed, post hoc analyses were performed with Newman–Keuls tests. Data for the minimal Feret’s diameter and the proportion of different type fibers were analyzed using mixed-effects linear models, and multiple comparisons were performed using diagonal contrasts. All statistical analyses were performed using the SAS software version 9.4, with the level of significance set at *p* < 0.05. The results are shown as means and standard error of the mean (SEM).
